# Biosynthesis and release of pheromonal bile salts in mature male sea lamprey

**DOI:** 10.1186/1471-2091-14-30

**Published:** 2013-11-04

**Authors:** Cory O Brant, Yu-Wen Chung-Davidson, Ke Li, Anne M Scott, Weiming Li

**Affiliations:** 1 Department of Fisheries and Wildlife, Michigan State University, Room 13 Natural Resources Building, 480 Wilson Road, 48824 East Lansing, MI, USA

**Keywords:** Pheromone, Gill, Bile salt, CYP7A1, 3-keto petromyzonol sulfate, Petromyzon marinus

## Abstract

**Background:**

In vertebrates, bile salts are primarily synthesized in the liver and secreted into the intestine where they aid in absorption of dietary fats. Small amounts of bile salts that are not reabsorbed into enterohepatic circulation are excreted with waste. In sexually mature male sea lamprey (*Petromyzon marinus* L.) a bile salt is released in large amounts across gill epithelia into water where it functions as a pheromone. We postulate that the release of this pheromone is associated with a dramatic increase in its biosynthesis and transport to the gills upon sexual maturation.

**Results:**

We show an 8000-fold increase in transcription of *cyp7a1*, a three-fold increase in transcription of *cyp27a1*, and a six-fold increase in transcription of *cyp8b1* in the liver of mature male sea lamprey over immature male adults. LC–MS/MS data on tissue-specific distribution and release rates of bile salts from mature males show a high concentration of petromyzonol sulfate (PZS) in the liver and gills of mature males. 3-keto petromyzonol sulfate (3kPZS, known as a male sex pheromone) is the primary compound released from gills, suggesting a conversion of PZS to 3kPZS in the gill epithelium. The PZS to 3kPZS conversion is supported by greater expression of *hsd3b7* in gill epithelium. High expression of *sult2b1* and *sult2a1* in gill epithelia of mature males, and tissue-specific expression of bile salt transporters such as *bsep*, *slc10a1*, and *slc10a2*, suggest additional sulfation and transport of bile salts that are dependent upon maturation state.

**Conclusions:**

This report presents a rare example where specific genes associated with biosynthesis and release of a sexual pheromone are dramatically upregulated upon sexual maturation in a vertebrate. We provide a well characterized example of a complex mechanism of bile salt biosynthesis and excretion that has likely evolved for an additional function of bile salts as a mating pheromone.

## Background

The *de novo* synthesis of bile salts from cholesterol primarily occurs in the liver of vertebrates in a series of reactions catalyzed by over 14 enzymes. Bile salts are secreted via the biliary system into the intestine where they facilitate absorption of lipids. Small amounts (roughly 5%) of these bile salts that are not returned to the liver via enterohepatic circulation are excreted from the body with waste [1-3]. Known additionally for their regulatory function, the enterohepatic circulation and metabolism of bile salts maintains cholesterol homeostasis and minimizes the cytotoxic effects of these compounds [2,4]. Studies of the sea lamprey (*Petromyzon marinus* L.), a member of the extant phylum Chordata, superclass Agnatha, have shown yet another function of bile salts – acting as pheromones that aid in chemical communication among conspecifics [5,6].

Dramatic alterations in the synthesis and excretion route of bile salts occur in the sea lamprey throughout its life history [7]. Upon reaching the reproductive stage, sea lamprey no longer feed, and the intestinal tract becomes highly atrophied [8]. Male sea lamprey have been shown to secrete bile salts and steroids through gill epithelia after sexual maturation [9]. Three of these compounds have been identified as 3-keto allocholic acid (3kACA), 3-keto petromyzonol sulfate (3kPZS), and petromyzestrosterol [5,10,11]. 3kPZS has been shown to attract sexually mature females to the odorant source in streams, acting as a sex pheromone [5,6,12].

In larval sea lamprey, bile salts are excreted through the urogenital pore. The filter-feeding larvae residing in streams excrete a lamprey-specific bile acid petromyzonol sulfate (PZS), and its putative precursor allocholic acid – ACA [13,14], as metabolic by-products with their feces. Both compounds are similar in structure to 3kACA and 3kPZS, respectively, but contain a hydroxyl group in place of the keto group at carbon-3 (C-3) position. Sorensen et al. [15] identified two additional larval compounds: petromyzonamine disulfate (PADS) and petromyzosterol disulfate (PSDS). Each compound examined (3kPZS, PZS, ACA, PADS, PSDS, and 3kACA) has been shown to stimulate the olfactory epithelium of migratory adults in electro-olfactogram recordings [5,14-16].

During a sea lampreys transformation from larval to adult life stage, complex and specific regulation of biosynthesis, transport, and secretion is likely required to promote physiologic functions [7]. We reasoned that sea lamprey have evolved physiological adaptations whereby conversion and excretion of bile salts are modified across life stages to exert various functions. We hypothesized that the life stage-specific secretion of pheromonal bile salts from mature male sea lamprey is due to changes in the biosynthetic and transportation pathway. In this study we present evidence of dramatic upregulation of bile salt synthesis in the liver of male sea lamprey after sexual maturation, transportation of these compounds to the gills via the bloodstream, and additional modification of PZS to 3kPZS in gill epithelia before secretion of 3kPZS into the environment as a mating pheromone.

## Results

### Tissue distribution of bile salts

**Figure 1:**
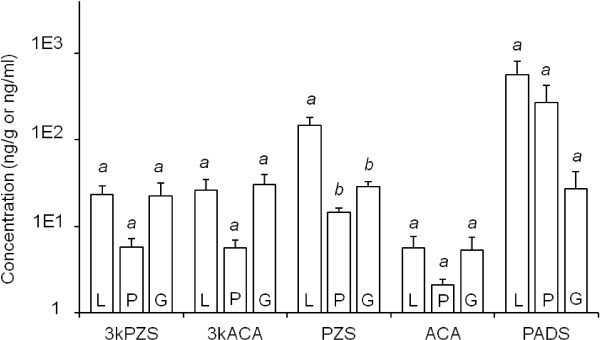
**Tissue-specific quantification of bile salts from immature male sea lamprey (*N*** **= 6).** 3-keto petromyzonol sulfate (3kPZS), 3-keto allocholic acid (3kACA), petromyzonol sulfate (PZS), allocholic acid (ACA), and petromyzonamine disulfate (PADS) were quantified in liver (L) and gill (G) tissues (ng/g), and plasma (P; ng/ml), using LC-MS/MS. Different lower-case letters indicate statistically significant differences between vertical columns within each group (compound). Vertical columns indicate means + 1 SEM, and were compared using ANOVA and post-hoc Tukey’s HSD (α = 0.05).

There are relatively minor variations in the distribution of bile salts across liver, plasma, and gills of immature males (IM). In IM, PADS was the most abundant compound detected, and its concentrations did not differ across tissues and plasma samples (ANOVA: *F*_2, 15_ = 2.75, *P* = 0.096). In IM, PADS was detected at 568.4 ± 238.3 ng/g (mean ± SEM, herein) in liver, 269.1 ± 152.2 ng/l in plasma, and 27.2 ± 15.7 ng/g in gill. PZS was the second most abundant compound detected, with concentrations that varied across tissues and plasma (*F*_2, 15_ = 16.08, *P* < 0.001). Mean concentration of PZS in the liver of 148.2 ± 31.4 ng/g was greater than mean plasma or gill concentrations of 14.5 ± 1.9 ng/l and 28.8 ± 3.8 ng/g, respectively (Tukey’s HSD, α = 0.05). 3kPZS, 3kACA, and ACA mean concentrations were lower and did not differ within each compound across liver, plasma or gills of IM (*F*_2, 15_ = 2.45, *P* = 0.120; *F*_2, 14_ = 3.23, *P* = 0.068; *F*_2, 14_ = 1.40, *P* = 0.279, respectively; Figure 1).

**Figure 2:**
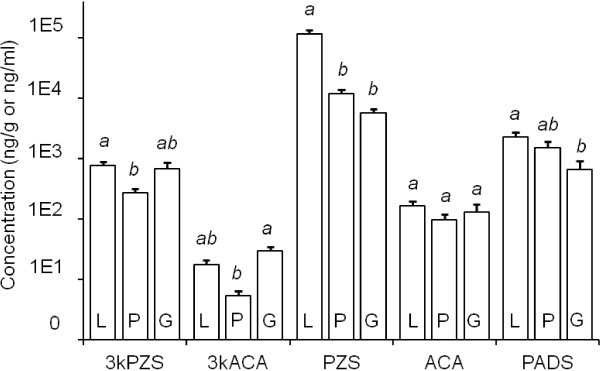
**Tissue-specific quantification of bile salts from sexually mature male sea lamprey (*N*** **= 12).** 3-keto petromyzonol sulfate (3kPZS), 3-keto allocholic acid (3kACA), petromyzonol sulfate (PZS), allocholic acid (ACA), and petromyzonamine disulfate (PADS) were quantified in liver (L) and gill (G) tissues (ng/g), and plasma (P; ng/ml), using LC-MS/MS. Subjects were a random sub-sample of those used for washings (see Figure 3). Different lower-case letters indicate statistically significant differences between vertical columns within each group (compound). Vertical columns indicate means + 1 SEM (vertical bars), and were compared using ANOVA and post-hoc Tukey’s HSD (α = 0.05).

In sexually mature males (SM), there are pronounced variations in the distribution of bile salts across liver, plasma and gills. The most abundant compound detected in SM was PZS. There were significant differences in PZS concentrations across tissues and plasma (*F*_2, 32_ = 26.6, *P* < 0.001). Mean concentration of PZS was highest in liver tissues of SM at 113401.1 ± 19784.8 ng/g, which was higher than a mean plasma concentration of 11678.6 ± 2089.7 ng/l-plasma) and mean gill concentration of 5604.5 ± 1028.4 ng/g, respectively (Tukey’s HSD, α = 0.05). Mean concentrations of bile salts differed across tissues and plasma for 3kPZS (*F*_2, 32_ = 4.26, *P* = 0.023), 3kACA (*F*_2, 27_ = 11.11, *P* < 0.001), and PADS (*F*_2, 32_ = 4.16, *P* = 0.025); however, concentrations of ACA did not differ across tissues and plasma (*F*_2, 32_ = 0.88, *P* = 0.424; Figure 2).

There were also dramatic differences in tissue distributions of bile salts between IM and SM (Table 1). Concentrations of PZS and 3kPZS were greater in liver, gill, and plasma of SM compared to IM. Compound ACA was detected in greater concentrations within liver and plasma of SM, compared to IM; however, there was no difference in gill concentrations of ACA between the two maturation stages. Compound PADS was detected in greater concentrations within liver of SM, compared to IM. There was no difference in gill or plasma concentrations of PADS between the two maturation stages. Concentrations of 3kACA were not different within liver, gill, and plasma of SM, compared to IM (Table 1).

**Table 1:** Comparison of bile salt distribution in tissues and plasma between immature male (IM) and sexually mature male (SM) sea lamprey

	**IM vs. SM**	
Compound - Tissue	*P*-value (*F*_df1, df2_)	
3kPZS - Gill	**0.017 (7.02**_**1, 16**_**)**	**IM < SM**
3kPZS - Liver	**< 0.001 (18.72**_**1, 16**_**)**	**IM < SM**
3kPZS - Plasma	**0.002 (14.08**_**1, 15**_**)**	**IM < SM**
3kACA - Gill	0.930 (0.01_1, 16_)	
3kACA - Liver	0.306 (1.17_1, 10_)	
3kACA - Plasma	0.861 (0.03_1, 15_)	
PZS - Gill	**0.002 (14.25**_**1, 16**_**)**	**IM < SM**
PZS - Liver	**0.001 (15.89**_**1, 16**_**)**	**IM < SM**
PZS - Plasma	**0.001 (16.49**_**1, 15**_**)**	**IM < SM**
ACA - Gill	0.053 (4.36_1, 16_)	
ACA - Liver	**0.013 (7.99**_**1, 15**_**)**	**IM < SM**
ACA - Plasma	**0.009 (8.86**_**1, 15**_**)**	**IM < SM**
PADS - Gill	0.088 (3.30_1, 16_)	
PADS - Liver	**0.034 (5.41**_**1, 16**_**)**	**IM < SM**
PADS - Plasma	0.055 (4.34_1, 15_)	

**Figure 3:**
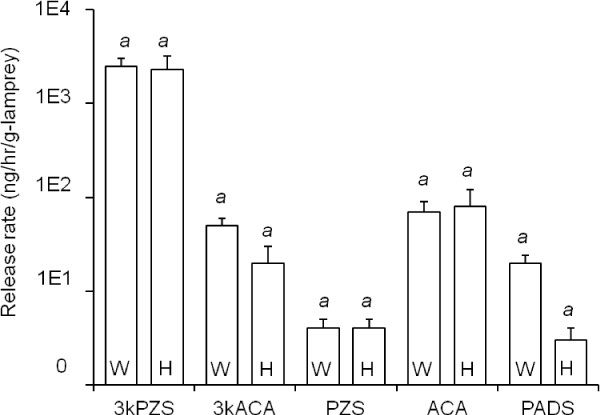
**Release rate (ng/hr/g-sea lamprey) of bile salts from sexually mature male sea lamprey.** 3-keto petromyzonol sulfate (3kPZS), 3-keto allocholic acid (3kACA), petromyzonol sulfate (PZS), allocholic acid (ACA), and petromyzonamine disulfate (PADS) were quantified in wash-water samples taken from the whole (W) body (*N* = 14) and from just the head (H) region (*N* = 6). Different lower-case letters indicate statistically significant differences between vertical columns within each group (compound). Vertical columns indicate means + 1 SEM (vertical bars), and were compared using ANOVA and post-hoc Tukey’s HSD (α = 0.05).

**Figure 4:**
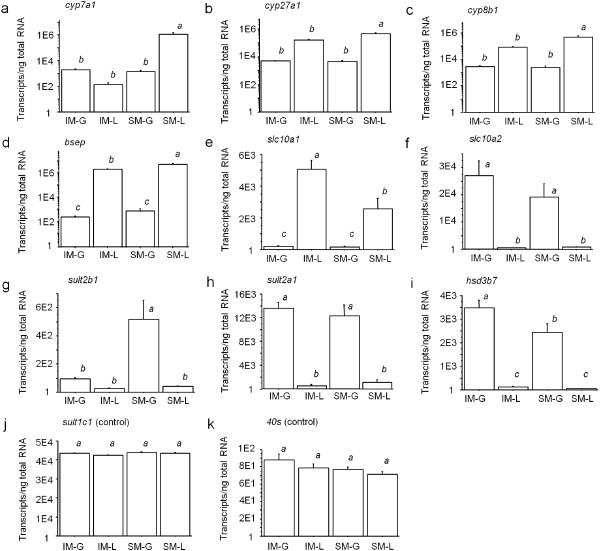
**Tissue-specific gene expression in sexually immature and mature sea lamprey.** Gill (G) and liver (L) tissues from immature males (IM, *n* = 15) and sexually mature males (SM, *N* = 15) were examined for each gene. Vertical columns represent mean transcripts/ng of total RNA ± 1 SEM. **a)***cyp7a1* mRNA, **b)***cyp27a1* mRNA, **c)***cyp8b1* mRNA, **d)***bsep* mRNA, **e)***slc10a1* mRNA, **f)***slc10a2* mRNA, **g)***sult2b1* mRNA, **h)***sult2a1* mRNA, **i)***hsd3b7* mRNA, **j)***sult1c1* mRNA standard, **k)***40s* ribosomal RNA standard. Different lowercase letters indicate statistically significant differences in mRNA abundance across tissues or maturities for each gene (ANOVA and post-hoc Tukey’s HSD; α = 0.05). Notice that the mRNA copy numbers (Y-axis) are expressed as exponents (E) of 10.

The compound and specific tissue compared between IM and SM are listed. Compounds include 3-keto petromyzonol sulfate (3kPZS), 3-keto allocholic acid (3kACA), petromyzonol sulfate (PZS), allocholic acid (ACA), and petromyzonamine disulfate (PADS). Actual concentrations can be seen in Figure 3 and 4. Bold font indicate significant differences between IM and SM (ANOVA and post-hoc Tukey’s HSD; α = 0.05).

### Release rate of bile salts

Sexually mature males released bile salts at substantially greater rates compared to immature males. Compounds 3kPZS, PZS, 3kACA, ACA, and PADS are released from the gills of SM, confirming previous findings for 3kPZS [9], and 3kACA [17]. Compound 3kPZS was released at the highest mean rate at 2483.0 ± 536.9 ng/g-body weight/hr (calculated from whole body washings) and 2272.8 ± 885.9 ng/g-body weight/hr (from washings taken from head region only). Release rates of ACA, 3kACA, PADS, and PZS ranged between 30–800 times lower than 3kPZS. There was no significant difference between whole body and head only release rates of 3kPZS (*F*_1, 18_ = 0.043, *P* = 0.837), 3kACA (*F*_1, 18_ = 1.90, *P* = 0.185), PZS (*F*_1, 18_ = 0.003, *P* = 0.956), ACA (*F*_1, 18_ = 0.044, *P* = 0.836), or PADS (*F*_1, 18_ = 3.46, *P* = 0.079; Figure 3. In SM tail washings; 3kPZS, ACA, 3kACA, PADS, and PZS were detected in minute concentrations (*e.g.* release rates were less than 0.7 ± 0.2 ng/g-body weight/hr). These results combined indicate that all detected bile salts were mainly released from the head region of SM.

The release rates of all tested compounds from head, tail, or full body washings of IM were below 0.5 ng/g-body weight/hr. Compounds detected (3kPZS, PZS, 3kACA, ACA, PADS) were released within the range of 0.404 and 0.005 ng/g-body weight/hr. Larval compound PSDS, known to stimulate the olfactory epithelium of migratory adult conspecifics [15], was not detected in SM or IM tissues or wash-water during this study suggesting that this compound is unique to larval sea lamprey. None of the six bile salts examined were detected in control washings across all chamber types and maturation stages.

### Tissue and maturation-specific gene expression

The three genes examined that encode for members of the cytochrome P450 monooxygenases were expressed at the highest level in the liver tissue of SM. Specifically, c*yp7a1* was expressed over 8000-fold greater in the liver tissue of SM compared to liver of IM and over 600-fold greater compared to gill tissues of IM and SM (*F*_3,56_ = 10.98, *P* <0.0001; Figure 4a). *Cyp27a1* was expressed greater than threefold in the liver tissue of SM compared to liver of IM and greater than 90-fold compared to gill tissues of IM and SM (*F*_3,56_ = 16.93, *P* <0.0001; Figure 4b). *Cyp8b1* was expressed six-fold greater in the liver tissue of SM compared to liver of IM and over 160-fold greater compared to gill tissues of IM and SM (*F*_3,56_ = 16.53, *P* <0.0001; Figure 4c). Expression of genes that encode for the bile salt export pump and sodium/bile salt cotransporters suggested that bile salt was transported from the liver to the gill. *Bsep* showed greater expression in the SM liver at over twofold that of IM liver and over 7000-fold that of SM and IM gill (*F*_3,51_ = 9.52, *P* < 0.0001; Figure 4d). *Slc10a1* was expressed greater than 13-fold in IM and SM liver compared to gills of both maturities (*F*_3,56_ = 27.71, *P* <0.0001; Figure 4e), while *slc10a2* was expressed over 25-fold greater in the gills of IM and SM compared to liver (*F*_3,55_ = 13.46, *P* <0.0001; Figure 4f). Additional bile salt modification such as the conversion of the 3-keto group and sulfate conjugation in the gills was also supported. Both *sult2b1*and *sult2a1* were expressed greater than 10-fold in gills of SM compared to liver of SM and IM (*F*_3,56_ = 12.13, *P* <0.0001 and *F*_3,56_ = 42.12, *P* <0.0001, respectively; Figure 4g,h). *Hsd3b7* was expressed greater than 20-fold in SM and IM gill tissue compared to all other tissues and maturities (*F*_3,56_ = 45.99, *P* <0.0001; Figure 4i). References *sult1c1* mRNA (Figure 4j) and *40s* ribosomal RNA (Figure 4k) were not significantly different across liver and gill of both IM and SM (*F*_3,56_ = 1.56, *P* = 0.211 and *F*_3,56_ = 2.18, *P* = 0.101, respectively).

## Discussion

Our results provide evidence of a dramatic upregulation of biosynthetic enzymes *cyp7a1*, *cyp27a1*[1,2], and the bile salt pool, in the male liver after sexual maturation (*i.e.* from immature to sexually mature adults). The upregulation may be an adaptive mechanism that satisfies a specific need to synchronize reproduction at the final life stage. In addition, a high concentration of several bile salts, mainly PZS, were found in the cardiac blood suggesting that a large amount of bile salts are transported to the gills. A likely route of bile salt transport to the gills is through hepatic veins that carry blood from the liver directly to the heart, which in turn pumps blood through the gills [18]. This system of bile salt transport in mature sea lamprey appears different from larval sea lamprey that actively filter feed in stream environments. As a result of circulating a bile salt pool from intestine to liver – *i*.*e*. enterohepatic circulation which plays a large role in cholesterol homeostasis [2], larval sea lamprey lose 5% of bile salts primarily as metabolic by-products with their waste [19]. Adult sea lamprey have been hypothesized to use these larval compounds as migratory cues [15,20], although the behavioral function of specific larval components remain to be characterized [21]. In the case of adult sea lamprey that have ceased feeding for close to two months [8], bile salts are not needed for digestion at this stage. On the contrary, certain bile salts such as 3kPZS are used as sex pheromones in mature sea lamprey [5,6].

Our LC–MS/MS analyses and gene expression data suggest that PZS is converted into 3kPZS in the gill epithelia. The dramatic inverse of PZS to 3kPZS ratios from inside to outside the body is strong evidence for this final conversion. PZS is present in higher concentrations in the liver, plasma, and gill tissues compared to 3kPZS, yet 3kPZS is secreted into the environment at the greatest rate of bile salts analyzed. Furthermore, *hsd3b7* is dominantly expressed in the gill tissue of adult male sea lamprey. Taken together, these results support the mechanism whereby PZS is dehydrogenated at C-3 to form 3kPZS, a reaction catalyzed by enzyme HSD3B7 [22], in the gill tissue before secretion into the environment. Interestingly, both *sult2b1*and *sult2a1* were expressed substantially greater in the gill tissues compared to liver tissues of adult males. *Sult2b1*and *sult2a1* encode for enzymes that catalyze the sulfate conjugation of steroid hormones (SULT2B1) and bile acids (SULT2A1). Sulfation increases the solubility of these compounds and facilitates secretion into the aquatic environment [23].

Tissue-specific expressions of putative bile salt transporter genes suggest that these putative transporters may play a role in gill excretion of bile salts in both IM and SM sea lamprey. In these life stages, lamprey do not recycle bile salts through enterohepatic circulation because the developmental biliary atresia during metamorphosis has closed the exocrine biliary pole of hepatocytes which are reorganized into solid cords [24]. For male adults to excrete pheromones, transporters need to provide mechanisms for the bile salts to exit hepatocytes as well as to enter and to exit gill epithelial cells. Expression of *bsep*, which encodes for an ATP-dependent bile salt export pump (BSEP) that is known for secretions of bile salts out of the hepatocytes [25], remained substantially high in the liver of both maturation states. As expected, the *bsep* mRNA level specifically increased in the lamprey liver from IM to SM, corresponding with the up-regulation of bile salt synthesis seen in SMs. In contrast, *slc10a1,* which encodes for a cotransporter that is responsible for uptake of bile salts into hepatocytes from blood, decreased from IM to SM males. This decrease might be a means for SM lamprey to elevate the blood PZS level, driving the bile salt toward the gills and away from a futile cycle back to the liver. Interestingly, *slc10a2*, encoding for a cotransporter primarily responsible for uptake of bile salts by apical cells lining the lumen of the small intestine [26], is expressed at a high level in the gill of adult males. Transporter SLC10A2 has been shown to have a high affinity for 5α-bile alcohols such as 3kPZS in lamprey [27]. It is possible that this Na^+^/bile salt cotransporter may play a role in gill uptake of bile salts from circulation.

Although our gene expression data are consistent with LC–MS/MS data in supporting a hypothesis for the hepatic synthesis and gill excretion of bile salts in SM sea lamprey, several caveats need to be addressed in future studies. First, the function of BSEP and SLC10A1 in transporting 5α-bile alcohols needs to be confirmed. The specific cellular locations of transporters (including SLC10A2) in the liver and gill should also be examined. Second, the transporter(s) that transport bile salts into water from gill cells need to be identified. Third, the enzymes involved with synthesis of C24 bile alcohols warrant an extensive study. Lampreys are unusual in producing both C24 and C27 bile alcohols as all other non-bony fish produce only C27 bile alcohols [28]. It is likely that the enzymatic mechanisms to cleavage side chains of cholesterol in producing C24 bile alcohols in sea lamprey is different from the multiple peroxisomal enzymes that produce C24 bile salts in human and rodent [28]. Once identified, the genes encoding these lamprey enzymes are expected to change in parallel with *cyp7a1* and *cyp8b1*, both of which were found to increase dramatically from IM to SM lamprey.

## Conclusions

**Figure 5:**
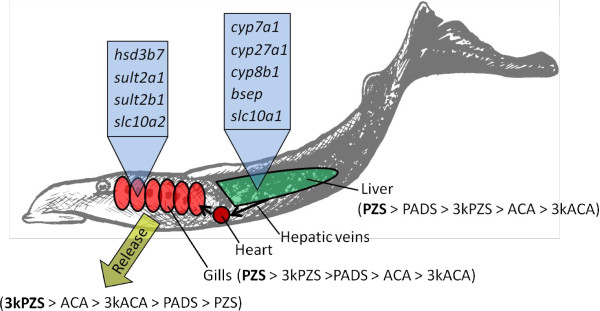
**Schematic of sexually mature male sea lamprey showing tissue-specific bile salt distribution and release.** Bile salts are shown in order of most abundant (bold font) to least abundant, and include: 3-keto petromyzonol sulfate (3kPZS), 3-keto allocholic acid (3kACA), petromyzonol sulfate (PZS), allocholic acid (ACA), and petromyzonamine disulfate (PADS). Genes examined that encode for enzymes associated with biosynthesis and transport of bile salts are shown in the respective tissues where they were greatest expressed, and include: *cyp7a1*, *cyp27a1*, *cyp8b1*, *bsep*, and *slc10a1* greatest expressed in liver tissue, and *hsd3b7*, *sult2a1*, *sult2b1* and *slc10a2* greatest expressed in the gill.

We show for the first time that a dramatic upregulation of *cyp7a1*, *cyp27a1*, and *cyp8b1* occurs primarily in the liver of male sea lamprey after reaching sexual maturation. Higher expression of *hsd3b7*, *sult2b1*, and *sult2a1* in gills of adult male sea lamprey suggest that additional bile salt modification occurs in gill tissues that may be associated with the conversion of PZS to 3kPZS. Our LC–MS/MS data on tissue distribution and release rates of bile salts support the theory of PZS to 3kPZS conversion in gills (Figure 5). We provide evidence for an example of a complex mechanism of bile salt biosynthesis and excretion that has been evolved to endow an additional function of a bile salt as a potent mating pheromone.

## Methods

### Sea lamprey collection and maintenance

All handling and dissections of sea lamprey were conducted in accordance with protocols approved by the Michigan State University Institutional Animal Care and Use Committee (AUF# 05-09-088-00). Sea lamprey were captured by the United States Fish and Wildlife Service and Fisheries and Oceans Canada using traps placed near dams in tributaries to Lake Michigan, Lake Superior, and Lake Huron. All captured lamprey were brought to the United States Geological Survey - Hammond Bay Biological Station (HBBS) and held in 500–1000 l aerated tanks fed continuously with Lake Huron water. The tank temperatures were kept between 17-19°C, which is similar to stream water temperature during a typical sea lamprey spawning season [8].

Immature male sea lamprey were visually identified and separated from females by carefully applying pressure to the lower abdomen to feel for eggs. Male sea lamprey were transferred to steel holding cages, ranging from approximately 0.25–1.0 m^3^ in volume, in the lower Ocqueoc River (at US23 bridge, Millersburg, MI, USA) to promote natural maturation in a spawning stream. Five acclimation cages, each containing roughly 10–15 male sea lamprey, were checked daily for signs of sexual maturation. Sexually mature males were identified by secondary sexual characteristics [29] including a pronounced rope-like ridge that develops dorsally along the length of the back to the anterior dorsal fin (see Additional file 1), and gamete release following gentle pressure applied to the lower abdomen. Acclimation time typically lasted 5–10 days before animals became sexually mature.

### Sea lamprey-conditioned wash water collection

Wash water samples from each male sea lamprey were collected for later quantification of bile salts. A bisected chamber was constructed following Siefkes et al. [9], with slight modification. Lamprey-conditioned water was collected from the anterior and posterior region of an individual, independently. Once an animal was secured into the bisected chamber, 3.5 l of deionized (DI) water was added to the posterior chamber, and the dividing air space was inspected for leaks. DI water (3.5 l) was then added to the anterior chamber. An additional non-bisected washing chamber (see Additional file 2) was used to confirm that chamber type did not alter release rates of compounds of interests. New individuals were transferred to a 20-l capacity container containing 5 l of DI water. A portable aerator was used for a constant supply of oxygen. The temperature of the DI water used for washings was acclimated to the same temperature as the holding tanks (17-19°C). Each chamber type was aerated while an individual was washed for 1 hr. Controls were conducted during each type of washing. For controls, all washing procedures remained the same excluding the addition of a lamprey to the chamber.

After washing, one 1 l and three 50 ml samples were taken from each chamber (head, tail, or whole body). All 1 l samples were immediately spiked with a 100 μl methanol 5-deuterated 3kPZS solution ([^2^H_5_]3kPZS; 5 ng/ml) that was custom synthesized by Bridge Organics Inc., Vicksburg, MI, as an internal standard (denoted as: 3kPZS-d_5_ IS, herein). After thorough mixing, the samples were stored at -20°C. All 50-ml samples were transferred directly to a -20°C freezer.

### Plasma and tissue collection

Males removed from both washing chamber types were euthanized with an overdose of tricaine methanesulfonate (200 mg/l MS222; Sigma-Aldrich, St. Louis, MO, USA). Blood was drawn directly from the heart using a 10 ml syringe (0.8 × 40 mm needle; BD SafetyGlide, Franklin Lakes, NJ, USA). Roughly 1.5 ml of blood from each male was transferred to a 1.5 ml Eppendorf Snap-Cap Microcentrifuge tube and centrifuged at 1500 rpm for 15 min at 4°C. Plasma supernatant was frozen at -80°C for later analyses. Two subsamples (20–50 mg) of each liver and gill, from consistent locations, were transferred to separate 2 ml tubes, snap-frozen in liquid nitrogen, and stored at -80°C for real-time quantitative PCR analyses. The remaining liver and gill were frozen at -20°C for bile salt distribution analyses.

### Measurement of bile salt distribution in washings and tissues by LC–MS/MS

Quantification of bile salts from washing samples followed methods by Li et al. [30] with slight modification. Each 1-l washing sample was thawed at room temperature and filtered twice with a glass microfiber filter of 1.0 μm nominal pore size (Whatman, Piscataway, NJ, USA) and once through a metrical grid filter of 0.45 μm pore size (Poll Corporation, Ann Arbor, MI, USA). Solid phase extraction (SPE) was accomplished by passing each filtered sample through a methanol-activated (2 ml of 100% MeOH) Oasis MCX mixed-mode polymetric sorbent cartridge (6 cc/500 mg; Waters, Milford, MA, USA). Eluants in methanol (8 ml/sample) were evaporated using a CentriVap Cold Trap with CentriVap Concentrator (Labconco, Kansas City, MO, USA). Residues were reconstituted with 100 μl of 50% (in DI water, vol:vol) high performance liquid chromatography (HPLC) grade methanol (Fisher Scientific, Fair Lawn, NJ, USA). Bile acid components were then identified and quantified using a Waters ACQUITY LC System coupled with the Waters Quattro Premier XE tandem quadrupole mass spectrometer (Milford, MA, USA).

100 μl of 5 ng/ml 3kPZS-d_5_ IS (in 75% ethanol, vol:vol with DI water) was added to adult immature male tissue samples, and 100 μl of 100 ng/ml 3kPZS-d_5_ IS (75% ethanol) was added to sexually mature male tissue samples. Ethanol (75%, vol:vol with DI water) was then added to each sample until the total volume reached 1 ml/100 mg of tissue. Tissues were homogenized and subjected to 15 hr of shaking at room temperature (~100 rpm). Each sample was then centrifuged at 13000 rpm for 10 min at room temperature. Supernatants were transferred to new vials and evaporated (Labconco). Residues were re-constituted in 1 ml of deionized water for SPE. SPE procedures followed those of washing samples using Oasis MCX cartridges. Eluants (8 ml each) were evaporated (Labconco) to a dry powdered state. Residues were reconstituted with 100 μl of 1:1 methanol:DI water (vol:vol) before injection into LC–MS/MS. Concentrations of each compound in each tissue was then standardized by the initial weight of each tissue sample (ng/g-tissue).

Plasma samples were extracted according to Scherer et al. [31], with slight modification. 100 μl aliquots of plasma were transferred to 15 ml tubes. Each aliquot was spiked with 10 μl of 5 ng/ml 3kPZS-d_5_ internal standard (in 50% methanol:DI, vol:vol). For protein precipitation, plasma was mixed with 1 ml acetonitrile and vortexed for 1 min. After 15 min of centrifugation (15000 rpm), the supernatant was filtered through a polyvinylidene fluoride (PVDF) syringe filter (0.22 μm pore size, 4 mm diameter; Membrane Solutions, Plano, TX, USA) and evaporated to a dry powered state. The samples were re-dissolved in 1 ml of 1:1 methanol:DI water (vol:vol). After an additional centrifugation (15000 rpm, 15 min), 10 μl of the methanolic supernatant was subjected to LC–MS/MS analyses. In all samples, bile salts examined include: (1) 3kPZS, (2) 3kACA, (3) PZS, (4) ACA, (5) PSDS, and (6) PADS.

### Real-Time Quantitative PCR (RTQ-PCR)

RTQ-PCR followed the procedures described by Chung-Davidson et al. [32] and Yeh et al. [7], with slight modification. Briefly, total RNA was extracted from gill and liver tissues using TRIzol Reagent (Invitrogen, Carlsbad, CA, USA), treated with TURBO DNA-free kit (Applied Biosystems, Foster City, CA, USA), and then reverse-transcribed into cDNA using Moloney Murine Leukemia reverse transcriptase (Invitrogen) and random hexamers (Promega, Madison, WI, USA). RTQ-PCR was performed using the TaqMan minor groove binder system (Applied Biosystems). Amplification plots were analyzed on an ABI-Applied Biosystems 7900 real-time PCR thermal cycler (Michigan State University Research Technology Support Facility, East Lansing, Michigan, USA). Synthetic oligonucleotides were used as standards and ran on the sample plate. Gene transcripts examined in sea lamprey gill and liver included: (1) *cyp7a1*, (2) *cyp27a1*, (3) *cyp8b1*, (4) *bsep*, (5) *slc10a1*, (6) *slc10a2*, (7) *hsd3b7*, (8) *sult2b1*, (9) *sult2a1*, (10) *sult1c1* mRNA, and (11) *40s* rRNA. The sequence information for each gene is listed in Table 2.

**Table 2:** Real-time quantitative PCR sequence information

**Gene ID**	**NCBI accession no.**	**Sequence for real-time quantitative PCR**
*cyp7a1*	PMZ0014378-RA	*CAACATGTCGGCGCTCATC*GC**CCTCCGAATACAACTCA**A*TGACACGCTGTCTCGCATG*
*cyp27a1*	PMZ0003691-RA	*TCTGGCCAAAATGTCATTCCT*T**AAGGCTGTCATCAAAG**AGATTC*TCAGACTGTATCCAGTGGTGCC*
*cyp8b1*	PMZ0014367-RA	*TCCCCTTGATAAAGGCCTCAT*CCCATGGCTGGGCCACGCCA**TCGAGTTCCGGAGGGA**C*ATGTACGCCTTCCTGCGG*
*bsep*	PMZ0007614-RA	*GTGTCTCAGGAGCCGGTGTT*G**TTCGACTGCAGCATTG**CCG*ACAACATTCGCTACGGTGCC*
*slc10a1*	PMZ0014657-RA	*CTGTCCCGGAGGGAACCT*CTCCAACGTGTTCGCGCTGGCGC**TCGACGGAGACATGAA**CCTC*AGCATCCTCATGACCACGTG*
*slc10a2*	PMZ0006503-RA	*GCTGGCGCTGGTGATGTT*CGCCATGGGCTGCA**CGGTGCAGATTCACAA**GGTGA*TTGCTCACCTTCGCAATCC*
*hsd3b7*	PMZ0008058-RA	*CGGCCGTTCTACAAGTTCGT*CCCACCCA**TCAACCGTCAGCTCGT**GG*TCATGGTCAACACGCACTTCA*
*sult2b1*	PMZ0018743-RA	*GGAACGTTCATGCGAAAAGG*CGCAG**TTGGCAACTGGAAAAG**T*GAATTCACCGTGGCGTTGA*
*sult2a1*	PMZ0001223-RA	*TGGTCACCTACCCCAAATCAG*GCACG**ACGTGGATGCAGGAGA**TCGTGACCCTGG*TGTATAGCGACGGGGACCTG*
*sult1c1*	PMZ0016497-RA	*CCGTGATGCGCGACAAC*CCCATGACG**AATTACAGCACCTTGCC**CACC*GACTTCTTGGACCACTCCGTG*
*40s*	PMZ0000703-RA	*ACCTACGCAGGAACAGCTATGAC*C**ATCTCGAGCAGCTGAA**GCTC*CAATGTGGTGGAATTCGTCG*

Partial sequences selected for amplification in RTQ-PCR experiments are shown, along with the NCBI accession number for each gene. Italicized sequences indicate the 5 and 3 primers for each gene. Sequences used for TaqMan MGB probes are shown in bold font. Note that the 3′ primer is the reverse compliment of the sequence shown above.

### Statistical analyses

All values from LC–MS/MS analyses associated with a signal-to-noise ratio ≤ 10 were considered below the lower limit of quantitation and automatically removed from the data set. All data presented were examined for violation of assumptions of normality and homogeneity across variance before further statistical analyses were conducted. Data that were not normally distributed or showed heterogeneity across variance were log-transformed. The Levene’s test for homogeneity of variance was used to examine variance of newly transformed data. Once homogeneity of variance was observed, an ANOVA and post-hoc Tukey’s HSD (α = 0.05) was conducted for statistical comparisons of LC–MS/MS data (R-Software® for Windows, R Foundation for Statistical Computing, Vienna, Austria), and for statistical comparisons of gene expression across tissues (StatView® for Windows, SAS Institute Inc., Cary, NC, USA).

## Competing interests

The authors declare that they have no competing interests.

## Authors’ contributions

COB contributed to the conception and design of this study, conducted all experiments involving animal treatments, collected and prepared all samples, performed real-time quantitative PCR, conducted all statistical analyses, and drafted the manuscript. YWCD conceived and designed molecular biology experiments and contributed to revision of the manuscript. KL performed all LC-MS/MS analyses, and contributed to design of chemistry experiments. AMS participated in RNA extraction, RTQ-PCR, and contributed to revision of the manuscript. WML conceived the overall study and contributed to writing of the manuscript. All authors read and approved this manuscript before submission.

## Supplementary Material

Additional file 1 **Nesting pair of sea lamprey (*Petromyzon marinus* L.).** Image shows a male (M) and female (F), as well as the male secondary sexual characteristic known as the rope (R). Scale bar = 20 mm. Click here for file

Additional file 2 Schematic of chambers used to collect lamprey-conditioned wash water. * = air stone. a) Bisected chamber capable of collecting washings from the head (h) and tail (t) region of the animal independently, following design by Siefkes et al. [9] with slight modification: the anterior end of the animal was inserted into a perforated acrylic tube (h), securing the body with an adjustable gasket. The mid-region of the animal was suspended across a dry space divider (s), assuring no possible leakage between h and t. The posterior was inserted into a perforated rubber tube (t), secured by an adjustable gasket. cm = centimeter. b) Basic 20 liter washing chamber that collected lamprey-conditioned water from the whole body (w) of the free-swimming animal. Click here for file
