# Supplementary figures and images for: Biosynthesis and release of pheromonal bile salts in mature male sea lamprey
Source: BMC Biochem. 2013 Nov 4;14:30. doi: 10.1186/1471-2091-14-30 (PMC3827326; doi:10.1186/1471-2091-14-30)

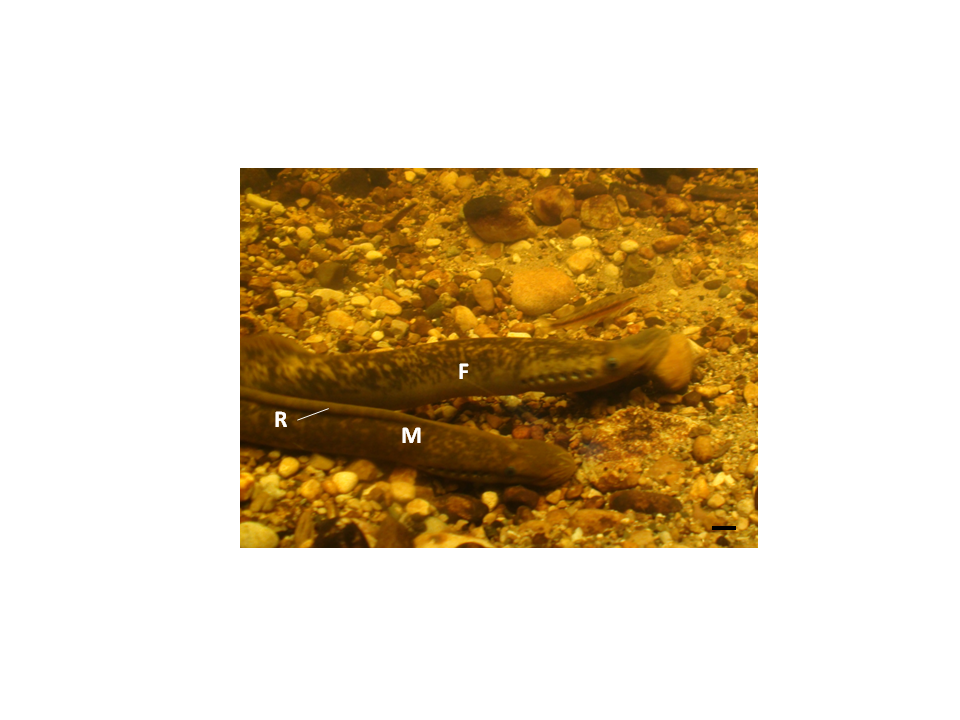

Supplement: Additional file 1 — Nesting pair of sea lamprey ( Petromyzon marinus L.). Image shows a male (M) and female (F), as well as the male secondary sexual characteristic known as the rope (R). Scale bar = 20 mm. [file 1471-2091-14-30-S1.png]
